# Patient-Level Fall Risk Prediction Using the Observational Medical Outcomes Partnership’s Common Data Model: Pilot Feasibility Study

**DOI:** 10.2196/35104

**Published:** 2022-03-11

**Authors:** Hyesil Jung, Sooyoung Yoo, Seok Kim, Eunjeong Heo, Borham Kim, Ho-Young Lee, Hee Hwang

**Affiliations:** 1 Office of eHealth Research and Business Seoul National University Bundang Hospital Seongnam-si Republic of Korea; 2 Kakao Healthcare Company-In-Company Seongnam-si Republic of Korea

**Keywords:** common data model, accidental falls, Observational Medical Outcomes Partnership, nursing records, medical informatics, health data, electronic health record, data model, prediction model, risk prediction, fall risk

## Abstract

**Background:**

Falls in acute care settings threaten patients’ safety. Researchers have been developing fall risk prediction models and exploring risk factors to provide evidence-based fall prevention practices; however, such efforts are hindered by insufficient samples, limited covariates, and a lack of standardized methodologies that aid study replication.

**Objective:**

The objectives of this study were to (1) convert fall-related electronic health record data into the standardized Observational Medical Outcome Partnership's (OMOP) common data model format and (2) develop models that predict fall risk during 2 time periods.

**Methods:**

As a pilot feasibility test, we converted fall-related electronic health record data (nursing notes, fall risk assessment sheet, patient acuity assessment sheet, and clinical observation sheet) into standardized OMOP common data model format using an extraction, transformation, and load process. We developed fall risk prediction models for 2 time periods (within 7 days of admission and during the entire hospital stay) using 2 algorithms (least absolute shrinkage and selection operator logistic regression and random forest).

**Results:**

In total, 6277 nursing statements, 747,049,486 clinical observation sheet records, 1,554,775 fall risk scores, and 5,685,011 patient acuity scores were converted into OMOP common data model format. All our models (area under the receiver operating characteristic curve 0.692-0.726) performed better than the Hendrich II Fall Risk Model. Patient acuity score, fall history, age ≥60 years, movement disorder, and central nervous system agents were the most important predictors in the logistic regression models.

**Conclusions:**

To enhance model performance further, we are currently converting all nursing records into the OMOP common data model data format, which will then be included in the models. Thus, in the near future, the performance of fall risk prediction models could be improved through the application of abundant nursing records and external validation.

## Introduction

Falls are the most commonly reported accidents that threaten patient safety in hospitals, particularly, because they may result in serious injuries—hip fractures and head injuries—or even death. Additionally, injurious falls increase hospital stays by up to 6 to 12 days and medical expenditures by $19,376 to $32,315 [1]. In 2015, the United States spent approximately $50 billion in fall-related additional medical costs [2]. However, most falls are considered preventable accidents, and since inpatient fall prevention depends on nursing quantity and quality, nurses have a key role.

Nurses periodically assess the risk of falls using screening tools such as the Hendrich II Fall Risk Model [3] and Morse Fall Scale [4] and provide additional nursing interventions. Furthermore, there have been ongoing attempts to improve the predictive performance of existing fall risk screening tools or develop a new prediction model altogether. Jung et al [5] improved fall prediction by integrating electronic health record data reflecting different types of data that were recorded over time and integrated from various sources. However, the participants were patients admitted to specific departments in a single hospital for a specific short period. One study [6] incorporated longitudinal electronic medical records and nursing data as covariates in calculating the fall risk and tested the model's performance through external validation. Nevertheless, the study had a limitation, in that, it did not comprehensively consider latent factors such as clinical test results. Marier et al [7] used structured electronic medical record data and the minimum data set to predict falls in nursing homes. These existing fall risk prediction models are limited because they were developed using small samples and limited covariates. Additionally, they lack standardized methodologies that allow their results to be reproduced by other researchers.

To overcome these limitations, Reps et al [8] proposed a standardized machine learning framework to generate and evaluate a clinical prediction model that leverages standardized clinical databases. Observational Health Data Science and Informatics (OHDSI) has created and applied an open-source data format and standardized analytics solutions to a diverse range of health and medical databases worldwide. The Observational Medical Outcome Partnership's (OMOP) common data model transforms heterogeneous source data into a common format using a set of common terminologies, vocabularies, and coding schemes. Thus, the OMOP common data model allows researchers to analyze health care big data from multiple sites consistently for development and replication [8]. In 2016, electronic health record data that included long-term care minimum data, drug dispensing data, and fall incident data from 5 skilled nursing facilities were converted into the OMOP common data model format [9]. Although the onset of major depressive disorder after beta-blocker therapy [10], symptomatic hemorrhagic transformation in patients with acute ischemic stroke [11], and cardioneurometabolic disease from full-night polysomnographic tests of patients [12] have been predicted using OMOP common data model data, there has been no attempt to predict inpatient fall risk in acute care settings using OMOP common data model data.

Therefore, the objectives of this study were to (1) convert fall-related electronic health record data into the standardized OMOP common data model data format and (2) develop a model that predicts fall risk at 2 risk time periods within 7 days of admission and during entire hospital stays, using OMOP common data model data as a pilot feasibility test.

## Methods

### Data Source

We used OMOP common data model data from Seoul National University Bundang Hospital, a tertiary general hospital located in a South Korean metropolis. Deidentified electronic health record data for more than 2 million patients who visited the hospital from May 2003 to July 2019 (patient demographic data, visit information, diagnoses, chief complaints, medications, test orders or results, interventions or surgeries, and family or past medical histories) were converted to the OMOP common data model format (version 5.3).

The OMOP common data model format consists of tables in which events of a different nature are stored. Signs, symptoms, and diagnosis are recorded in the *CONDITION_OCCURRENCE* table; activities or processes of a diagnostic or therapeutic nature ordered, or carried out by, a health care provider are recorded in the *PROCEDURE_OCCURRENCE* table; exposure to a drug (ingested or otherwise introduced into the body) are recorded in the *DRUG_EXPOSURE* table; clinical facts (including social and lifestyle facts and medical and family history) about patients obtained in the context of examination or interview are recorded in the *OBSERVATION* table; and orders and the results of laboratory tests, vital signs, and quantitative findings from pathology reports are recorded in the *MEASUREMENT* table. Events where persons or patients visit the health care system for a duration of time (for example, inpatient, outpatient, or emergency room visits) with detailed information are recorded in the *VISIT_OCCURENCE* and *VISIT_DETAIL* tables, respectively. All tables are linked to the *PERSON* table, which includes each person or patient and some demographic information, providing a person-centric relational data model [13].

### Study Population

The target population consisted of patients over 18 years admitted to neurology, neurosurgery, hematology, or oncology departments from January 1, 2010 to July 18, 2019 at Seoul National University Bundang Hospital. Accidental falls have most frequently occurred in these departments in this hospital. The outcome cohort consisted of patients who had experienced falls. Patients who had a fall were identified by using 9 structured and standardized statements. Since 2003, the Seoul National University Bundang Hospital has used standardized nursing statements, with a unique predefined code that is built on the International Classification for Nursing Practice, which have been validated in previous fall-related studies [5,14]. We also searched the free-text narratives that included the words or phrases “fall down,” “slip and fall,” and “collapsed” to identify patients who had falls but had not been highlighted by the 9 structured and standardized statements. To identify to which medical departments patients had been admitted, we used the specialty data of the physician (provider) who treated the patient by combining the *VISIT_OCCURRENCE* and *PROVIDER* tables, first, or care site data, obtained from the *VISIT_DETAIL* table. If there were no care site data in the *VISIT_DETAIL* table, we used ward information from the *visit_detail_source_value* field (Multimedia Appendix 1).

### Conversion of Fall-Related Electronic Health Record Data Into OMOP Common Data Model Format

Fall-related electronic health record data were converted into OMOP common data model format through an extraction, transformation, and load process. We extracted the data from the nursing notes, fall risk assessment, patient acuity assessment, and clinical observation sheets. Next, we integrated and standardized the data in the common data model format using standardized terminologies (SNOMED CT and LOINC). Nine structured nursing statement items and free-text narratives that included the words or phrases “fall down,” “slip and fall,” and “collapsed” in the nursing notes were manually mapped to 3 standard concepts (*SCTID = 33036003 |Fall on same level (event)|*, *SCTID = 242120009 |Fall on public service vehicle (event)|*, *SCTID = 20902002 |Fall from bed (event)|*) within SNOMED CT corresponding to the observation table according to the types of fall [5].

The Hendrich II Fall Risk Model total score and patient acuity score were mapped to *444514002 |Hospital falls risk assessment score for the elderly (observable entity)|* and *425705009 |Determination of acuity level (procedure)|* concepts, respectively, and loaded into the observation table. Clinical observation data such as vital signs, level of consciousness (for example, Glasgow Coma Scale score), volume of output (such as urine and fluid), and pain score were also manually mapped to standard concepts within the LOINC or SNOMED CT codes corresponding to the measurement and observation tables. The LOINC and SNOMED CT codes were identified in the OHDSI standard vocabulary to arrive at OHDSI concept identifiers.

**Figure 1:**
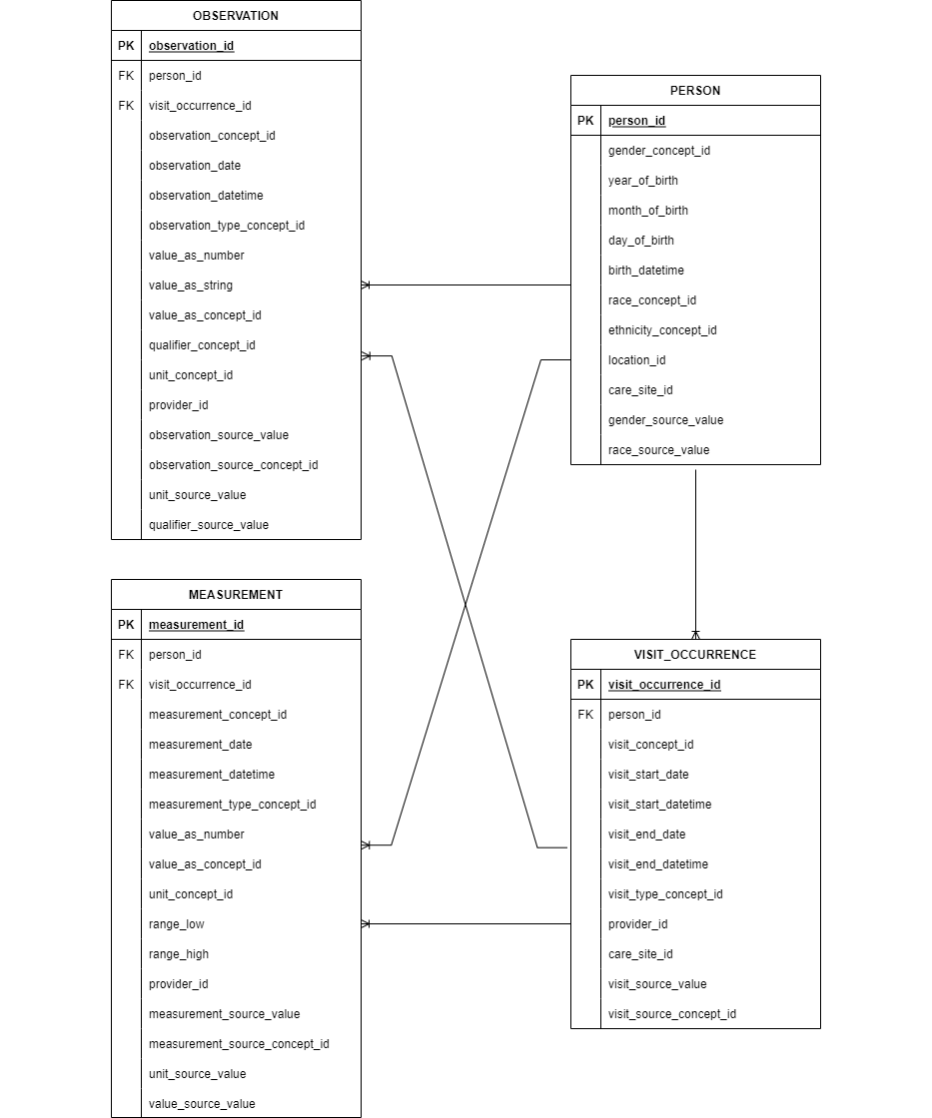
Relationship between common data model tables containing fall-related electronic health record data.

For internal and external validation, the mapping results of a nurse with abundant terminology mapping experience were reviewed and validated by a clinical terminologist. When both agreed on the mapping results, they were considered internally valid. If they disagreed, the results were discussed in group meetings attended by other researchers and domain experts (such as critical care nurses and clinical pathologists) who were not involved in the mapping process, but had experience of SNOMED CT or LOINC mapping. The measurement and observation tables were linked to *PERSON* and *VISIT_OCCURRENCE* tables based on their foreign keys (Figure 1). After completing the extraction, transformation, and load process, data quality was assessed by ACHILLES [13]. Finally, fall-related electronic health record data integrated into the existing common data model were utilized for the feasibility test.

### Fall Risk Prediction Using Open-Source OHDSI Analytic Tools

We utilized multiple covariates including sex, age (over 60 years of age or not), diagnoses, prescriptions, history of falling, values in laboratory tests or vital signs, interventions or surgical procedures, an atrial fibrillation stroke risk score (CHA2DS2-VASc—congestive heart failure, arterial hypertension, age over 75 years, diabetes, stroke or transient ischemic attack, vascular disease, age 65-74 years, sex), Diabetes Complications Severity Index, Charlson comorbidity score, patient acuity score, and visit count to train the prediction model.

The baseline characteristics of the study population with and without falls occurring during entire hospital stays from admission day onward were compared using 2-tailed independent sample *t* tests for continuous covariates and chi-square tests for categorical covariates. Furthermore, the observation periods used to construct covariates were set as 30 and 90 days prior to the admission date (excluding the day of admission). Data recorded more than 90 days prior to admission were not included when constructing the covariates, because we considered patients’ conditions at the time closest to admission to be most important in predicting the risk of falling.

The study population was randomly split into training (75%) and testing (25%) sets. We used least absolute shrinkage and selection operator–based logistic regression and random forest algorithms and selected optimal hyperparameters using 5-fold cross validation with the training set. For the evaluation of predictive performance, the area under the receiver operating characteristic curve (AUROC), sensitivity, specificity, and negative predictive value were calculated and compared to those from the Hendrich II Fall Risk Model from the same interval (within 7 days after admission or throughout the hospital stay). The total score ranges from 0 to 16, with the patient being considered at a high risk of falling when the total score is 5 or higher. Since the aim of assessing or predicting the risk of falling is to provide more effective preventive care, we gave greater weight to sensitivity and negative predictive value among the predictive indicators [14,15].

An open-source package (PatientLevelPrediction, version 4.0.5) and R software (version 4.0.3) were used to develop and evaluate the prediction model.

This study was approved by and performed in accordance with the relevant guidelines and regulations of the Seoul National University Bundang Hospital institutional review board (X-2106/689-902).

## Results

### Fall-Related Electronic Health Record Conversion Into OMOP Common Data Model

**Figure 2:**
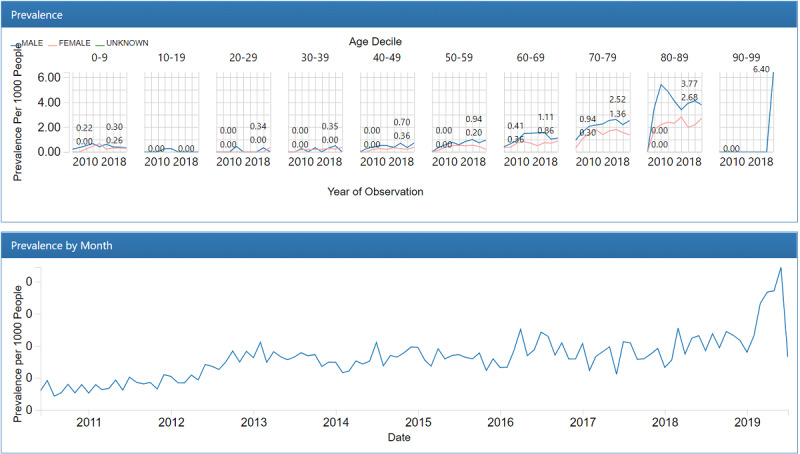
Descriptive common data model report for the fall from bed concept.

**Figure 3:**
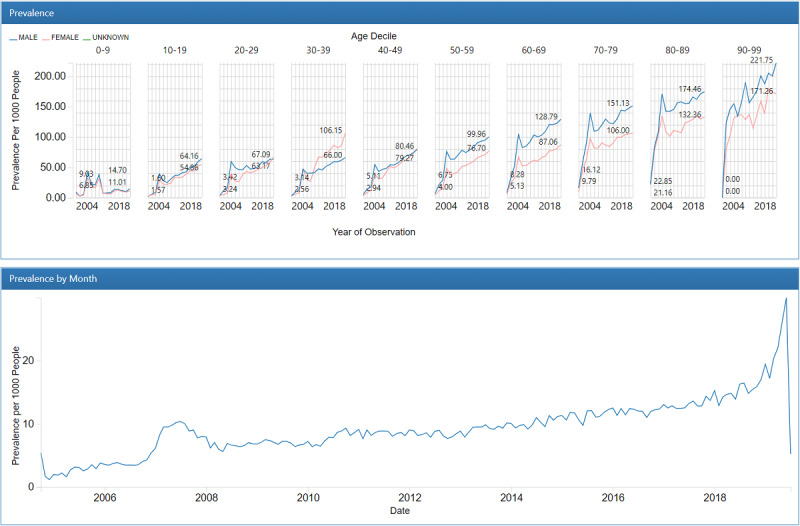
Descriptive common data model report (hospital falls risk assessment score for older adults).

Of the 385,272,691 nursing statements extracted from the nursing notes, 6277 records representing fall accidents were converted to the observation table in the OMOP common data model. We converted 747,049,486 records within the clinical observation sheet into the OMOP common data model, of which 84.3% (629,535,325 records) were represented by standard vocabularies (SNOMED CT: 64,008,725/747,049,486 8.6%; LOINC: 565,526,600/747,049,486, 75.7%), and 15.7% (117,514,161/747,049,486) were not (Table 1). A total of 1,554,775 Hendrich II Fall Risk Model total scores and 5,685,011 acuity scores were converted into the observation table in OMOP common data model. Sample descriptive reports (Figures 2 and 3) from the OHDSI ACHILLES data characterization program show the prevalence of concepts per 1000 people by sex, age group, and year.

**Table 1:** Fall-related electronic health record data standardization.

Electronic health record data, common data model domain, and standard vocabulary			Mapped items, n	Converted records, n
**Nursing statement**				
	**Observation**			
		SNOMED CT	9	6277
**Clinical observation sheet records**				
	**Measurement**			
		LOINC	199	520,381,084
		SNOMED CT	11	7,421,380
	**Observation**			
		LOINC	74	45,145,516
		SNOMED CT	18	56,587,345
**Fall risk score**				
	**Observation**			
		SNOMED CT	1	1,554,775
**Patient acuity score**				
	**Observation**			
		SNOMED CT	1	5,685,011

### Characteristics of the Study Population

A total of 109,289 inpatients were admitted to the neurology, neurosurgery, hematology, and oncology departments. Among them, 1465 patients fell during their hospitalization. In patients who had a fall, a larger proportion were aged 70-79 years; had previously fallen; had malignant neoplastic disease (individuals who had fallen: 753/1,465, 51.4%; individuals who had not fallen: 44,605/107,824, 41.4%), which was the most frequently observed condition medical history within 90 days before admission; and tended to take more central nervous system agents, such as antiepileptics, antidepressants, and antipsychotics than individuals who had not fallen. At the time of admission, the mean Hendrich II Fall Risk Model total scores for individuals who had and who had not fallen were 4.3 and 2.5 points, respectively, and patient acuity score for individuals who had fallen (mean 23.2 points) was higher than that of individuals who had not fallen (mean 19.8 points). The median duration of hospital stay for individuals who had not fallen was 4 days, whereas that for individuals who had fallen was 15 days (Table 2).

**Table 2:** Study population.

Characteristic			Fall (n=1465)		No fall (n=107,824)		*P* value
**Sex, n (%)**							<.001
	Male	845 (57.68)		56,369 (52.28)			
	Female	620 (42.32)		51,455 (47.72)			
**Age group (years), n (%)**							
	19-29	40 (2.73)		4339 (4.02)		.01	
	30-39	68 (4.64)		7368 (6.83)		<.001	
	40-49	141 (9.62)		14,457 (13.41)		<.001	
	50-59	231 (15.77)		25,033 (23.22)		<.001	
	60-69	388 (26.49)		26,857 (24.91)		.17	
	70-79	449 (30.65)		22,931 (21.27)		<.001	
	Over 80	148 (10.10)		6839 (6.34)		<.001	
Previous fall, n (%)			103 (7.03)		2143 (1.99)		<.001
**Condition, n (%)^a^**							
	Malignant neoplastic disease	753 (51.40)		44,605 (41.37)		<.001	
	Intracranial aneurysm	31 (2.12)		13,231 (12.27)		<.001	
	Neoplasm of head	234 (15.97)		9562 (8.87)		<.001	
	Diabetes	105 (7.17)		4328 (4.01)		<.001	
	Traumatic and nontraumatic brain injury	95 (6.48)		4435 (4.11)		<.001	
	Osteoarthritis	30 (2.05)		1042 (0.97)		<.001	
**Medication use, n (%)^a^**							
	Antiepileptics	392 (26.76)		15,639 (14.50)		<.001	
	Antidepressants	181 (12.35)		6969 (6.46)		<.001	
	Antipsychotics	188 (12.83)		6566 (6.09)		<.001	
	Vasoprotectives	813 (55.49)		47,284 (43.85)		<.001	
	Antihemorrhagics	197 (13.45)		9375 (8.69)		<.001	
**Procedure or operation, n (%)^a^**							
	Computed tomography of brain without contrast	216 (14.74)		8577 (7.95)		<.001	
	Magnetic resonance imaging of head and neck with contrast	86 (5.87)		5681 (5.27)		.34	
	Transfusion of platelet concentrate	105 (7.17)		4067 (3.77)		<.001	
**Measurement value, mean**							
	Percentage segmented neutrophils in blood	65.92		60.70		<.001	
	Heart rate	84.09		80.04		<.001	
	Glucose level (mg/dL in serum, plasma, or blood)	128.74		117.73		<.001	
Visit count, mean			13.31		10.69		<.001
Hendrich II Fall Risk Score, mean			4.29		2.48		<.001
Patient acuity score, mean			23.17		19.75		<.001
Length of stay (days), median			15		4		<.001

^a^Not applicable to all patients; therefore, percentages do not add to 100%.

### Predictive Performance

A total of 220 individuals who had fallen (0.81%) among 27,201 inpatients and 369 (1.36%) individuals who had fallen among 27,109 inpatients were identified to have fallen within 7 days of admission and during their entire stay, respectively, from testing set by time. The prediction feasibility test based on common data model data yielded AUROC values from 0.692 to 0.726. In general, our models showed better predictive performance than that of the Hendrich II Fall Risk Model, which used data recorded at admission or at the closest date to the admission date (Table 3). In calibration plots for the models (Multimedia Appendix 2), confidence intervals were wide due to the low frequency of falls; nevertheless, the predicted and observed risks tended to be proportional.

**Table 3:** Predictive performance.

Time point and algorithm		Outcome rate (%)	AUROC (95% CI)	Sensitivity (%)	Specificity (%)	Negative predictive value (%)
**Within 7 days of admission**						
	LASSO^a^ logistic regression	0.81	0.718 (0.686-0.750)	65.91	64.24	99.57
	Random forest		0.692 (0.661-0.724)	66.82	62.51	99.57
	Hendrich II Fall Risk Model	0.78	0.677 (0.658-0.696)	53.81	74.52	99.51
**During entire hospital stay**						
	LASSO logistic regression	1.36	0.726 (0.702-0.750)	68.29	63.43	99.31
	Random forest		0.723 (0.698-0.747)	69.11	62.87	99.33
	Hendrich II Fall Risk Model	1.35	0.673 (0.659-0.687)	52.43	74.05	99.13

^a^LASSO: least absolute shrinkage and selection operator.

### Variables

**Figure 4:**
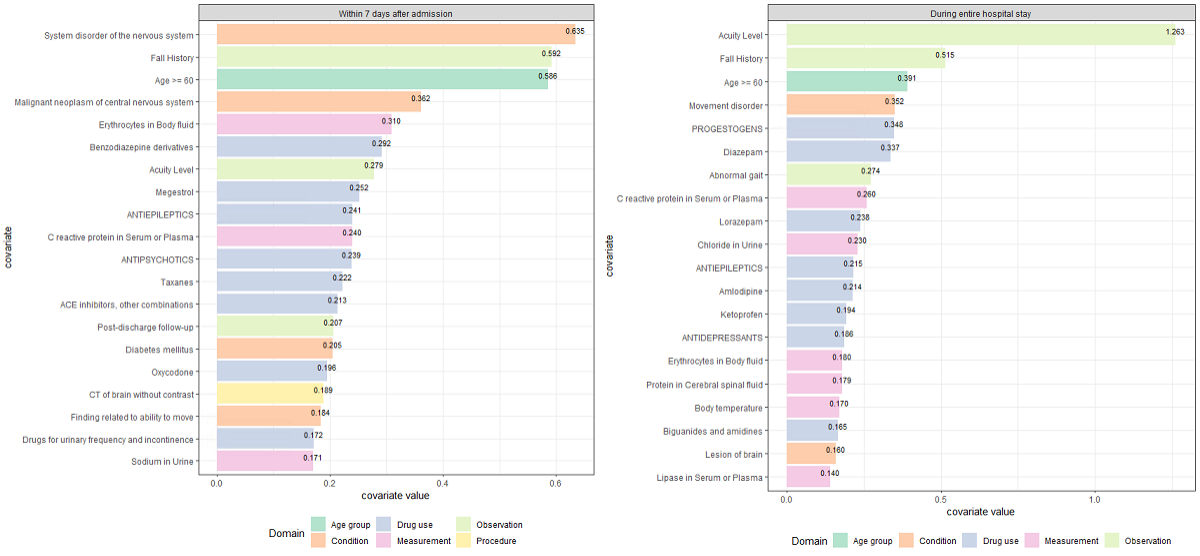
Top 20 covariates included in the logistic regression models by risk time period.

Of 13,405 candidate predictors, 103 and 154 covariates were included in the logistic regression models for 7 days after admission and for the entire hospital stay, respectively. Among the top 20 covariates in the logistic regression models (Figure 4), 6 were selected for both time models. Patients' acuity scores and fall histories were the most powerful in increasing the risk of falls in the logistic regression model. In addition, age over 60 years, antiepileptic medications, C-reactive protein in serum or plasma, and erythrocyte count in body fluid were identified as common important covariates (Multimedia Appendix 3).

## Discussion

### Principal Results

To the best of our knowledge, this study is the first attempt predicting inpatients’ fall risk in an acute care setting using OMOP common data model data. We converted fall-related nursing statements, fall risk, patient acuity scores, and clinical observation sheet records into OMOP common data model format using standard terminologies such as SNOMED CT and LOINC.

In the process of transforming fall-related electronic health record data to OMOP common data model format, we were able to map 50.0% (306/612) of the data items in the clinical observation sheet to the standard vocabularies, covering 84.3% (629,535,325/747,049,486) of the total clinical observation sheet records. The categories *drain* and *medication* were not mapped to the standard vocabularies. The *drain* category contained data items related to fluid output by tube type (for example, Jackson-Pratt drain, chest tube, jejunostomy, and external ventricular drain), and it is impossible to represent fluid output by tube type using predefined standard vocabularies. The *medication* category included items on volume of parenteral fluid input by drug type (such as dopamine, herben, epinephrine, and heparin); we could not map these to more detailed precoordinated concepts than *251855004 |Parenteral fluid input (observable entity)|*.

According to some studies [16,17], typically, more than 1000 patients with the outcome being studied are needed for developing a prediction model. We found that 1465 of 109,289 inpatients had experienced accidental falls during their hospital stays—an incidence of 1.34%. These figures are lower than those of previous studies—1.66% [6] and 3.50% [18]. Boyce et al [9] showed that there was a gap in the number of falls between data sources [9]; if we had included the fall incident report as a data source, individuals who had fallen who had not been recorded in the nursing notes could have been identified.

Nursing notes are valuable sources of data for inpatient fall research. One study [19] found that nursing notes had the highest coverage for features related to inpatients’ fall. For example, records of walking aid use, lower extremity strength, caregivers, fatigue, and sleep disturbance, which are important factors affecting the occurrence of falls, can only be extracted from nursing notes. Nevertheless, most nursing note records have yet to be converted into the OMOP common data model data format, and therefore, could not be used here. With the complete conversion of nursing records into the common data model data format in the near future, we expect that the performance of fall risk prediction models will be improved.

The AUROC, sensitivity, and negative predictive values were higher than those of Hendrich II Fall Risk Model assessed at the time of admission for the same patients. When we applied the best threshold (2.5 points) to Hendrich II Fall Risk Model, sensitivities improved to 62.46%-63.61% by risk time; however, this was still lower than the sensitivities (65.91%-69.11%) of the developed models. In this study, logistic regression had better predictive performance than that of random forest algorithms in terms of the AUROCs at both risk time periods, whereas the logistic regression showed similar or rather lower performance than that of random forest algorithms in terms of sensitivity and negative predictive value.

With respect to the possibility of applying prediction rules to other data, logistic regression is superior to the random forest method since the regression coefficients are known and can be applied [20]. Additionally, least absolute shrinkage and selection operator–based logistic regression models generally have greater parsimony than other machine learning models [11,21]; our study also showed this; thus, the least absolute shrinkage and selection operator–based logistic regression model is used as the reference algorithm for the OHDSI analytics pipeline for patient-level prediction.

### Comparison With Prior Work

The predictive performance of our models was higher than those of another study [18], while the AUROC values were lower than those of other studies [5,6], that also used nursing records. These studies [5,6] have critical limitations, in that, they used small samples (15,480 [5] and 14,307 [6] admissions) and excluded covariates related to clinical laboratory test results and visit count. In particular, Cho et al [6] may have overfitted by oversampling the individuals who had fallen by using the synthetic minority oversampling technique to eliminate the data imbalance between individuals who had fallen and individuals who had not fallen.

The patient acuity score was identified as the most important covariate (covariate value 1.263) for falling from the logistic regression model. Previous studies [5,22] also reported that individuals who had fallen had higher acuity scores compared to individuals who had not fallen. The patient acuity score is calculated based on clinical patient characteristics and nursing workload, which reflects patient dependency and severity. For example, the number of visits for oral medications and number of complicated intravenous drugs or transfusion are components of the patient acuity tool. Mobility or movement difficulty, which is an important factor for falling, is also reflected in the patient acuity score. Therefore, the patient acuity score could be an indicator of patients' physical vulnerability and could reflect fall risk.

Fall history was also identified as an important covariate (covariate value 0.515) for falling, which is consistent with the findings of systematic literature reviews [23,24]. American Geriatrics Society/British Geriatrics Society [25] and Australian [26] Clinical Practice Guidelines for the prevention of falls in older adults recommend that all patients be asked whether they have fallen previously because a history of falling is generally a good predictor of future falls. Additionally, having fallen within a 3-month period is one of variables of the Morse Fall Scale and Downton Fall Risk Index, which assesses a patient's likelihood of falling in an acute care setting. Patients who had experienced accidental falls feared falling [27]. It has been estimated that the prevalence of fear of falling in older adults is approximately 90% among those who had previously fallen compared to 65% among individuals who had not previously fallen [27,28]. Fear of falling in persons who have previously fallen may originate from a concern about falling, loss of balance, loss of confidence, and avoidance of activities.

The presence of movement disorders, such as abnormal gait, which has been consistently identified as a strong risk factor for falling by some systematic reviews [24,29], was also included in the top 20 covariates of fall risk prediction models using the least absolute shrinkage and selection operator–based logistic regression algorithm. Furthermore, central nervous system agents (antiepileptics and benzodiazepine derivatives, such as diazepam) were identified as predictors in this study. This result is consistent with that of a previous study [30] that suggested that psychotropic medications increased fall risk by 1.36-1.39 times in older adults. Interestingly, progestogens were identified as important predictors (covariate value 0.348) of falls that occurred for the entire hospital stay period. Although we cannot explain their causal relationship, it is well known that the levels of reproductive hormones such as estrogen and progesterone is related to the development of musculoskeletal disorders in women. As such, since some musculoskeletal disorders were identified as predictors of falls, progestogens may have indirectly influenced the occurrence of some of the falls.

### Limitations

The following limitations should be considered when interpreting the results of this study. First, the generalizability of the models remains low, since the basis of the models’ development was patients admitted to specific medical departments of a single hospital from 2010 to 2019. However, since the purpose of this study was to demonstrate the feasibility of the prediction model, the fall risk prediction model will be improved by adding all nursing records and applying external validation in the future. Second, the number of falls may have been underestimated, as we used only structured nursing statements to identify the occurrence of falls. Third, we only used logistic regression and random forest algorithms to develop fall prediction models, because regression-based algorithms perform better in smaller, single-center data sets [21,31] and have greater parsimony than other machine learning models [11]. In the future, we will work with multiple institutions to conduct OMOP common data model–based fall prediction research and apply other modern machine learning algorithms. Fourth, because the conversion of electronic health record data to standard vocabularies depends on the quality of the mapping tables or the medical coder’s (or terminologist’s) mapping skills [32], mapping results could differ depending on mapping purpose and institutions. Therefore, since standard vocabularies change constantly, all researchers and institutions utilizing OMOP common data model data should have in-depth understanding and training on standard terminologies.

### Conclusions

To the best of our knowledge, this is the first study to transform fall-related electronic health record data into OMOP common data model format and utilize the resulting data to develop fall risk prediction models for acute care settings. The performance of the developed models was superior to that of Hendrich II Fall Risk Model, which the study hospital uses to screen fall risk. Patient acuity score, history of falls, age over 60 years, movement disorder, and central nervous system agents such as psychotropic medications were identified as important covariates for fall risk prediction.

## Appendix

Multimedia Appendix 1 Detailed definitions of target and outcome cohorts.

Multimedia Appendix 2 Calibration plots.

Multimedia Appendix 3 Full list of covariates selected for logistic regression and random forest models by risk time period.

## Abbreviations

ACHILLES: Automated Characterization of Health Information at Large-scale Longitudinal Evidence Systems
AUROC: area under the receiver operating characteristics curve
LOINC: Logical Observation Identifiers Names and Codes
OHDSI: Observational Health Data Science and Informatics
OMOP: Observational Medical Outcome Partnership
SNOMED CT: Systematized Nomenclature of Medicine–Clinical Terms
