# Supplementary material for: Patient-Level Fall Risk Prediction Using the Observational Medical Outcomes Partnership’s Common Data Model: Pilot Feasibility Study
Source: JMIR Med Inform. 2022 Mar 11;10(3):e35104. doi: 10.2196/35104 (PMC8957002; doi:10.2196/35104)
Supplement: Multimedia Appendix 2 [file medinform_v10i3e35104_app2.pptx]

## Slide 1
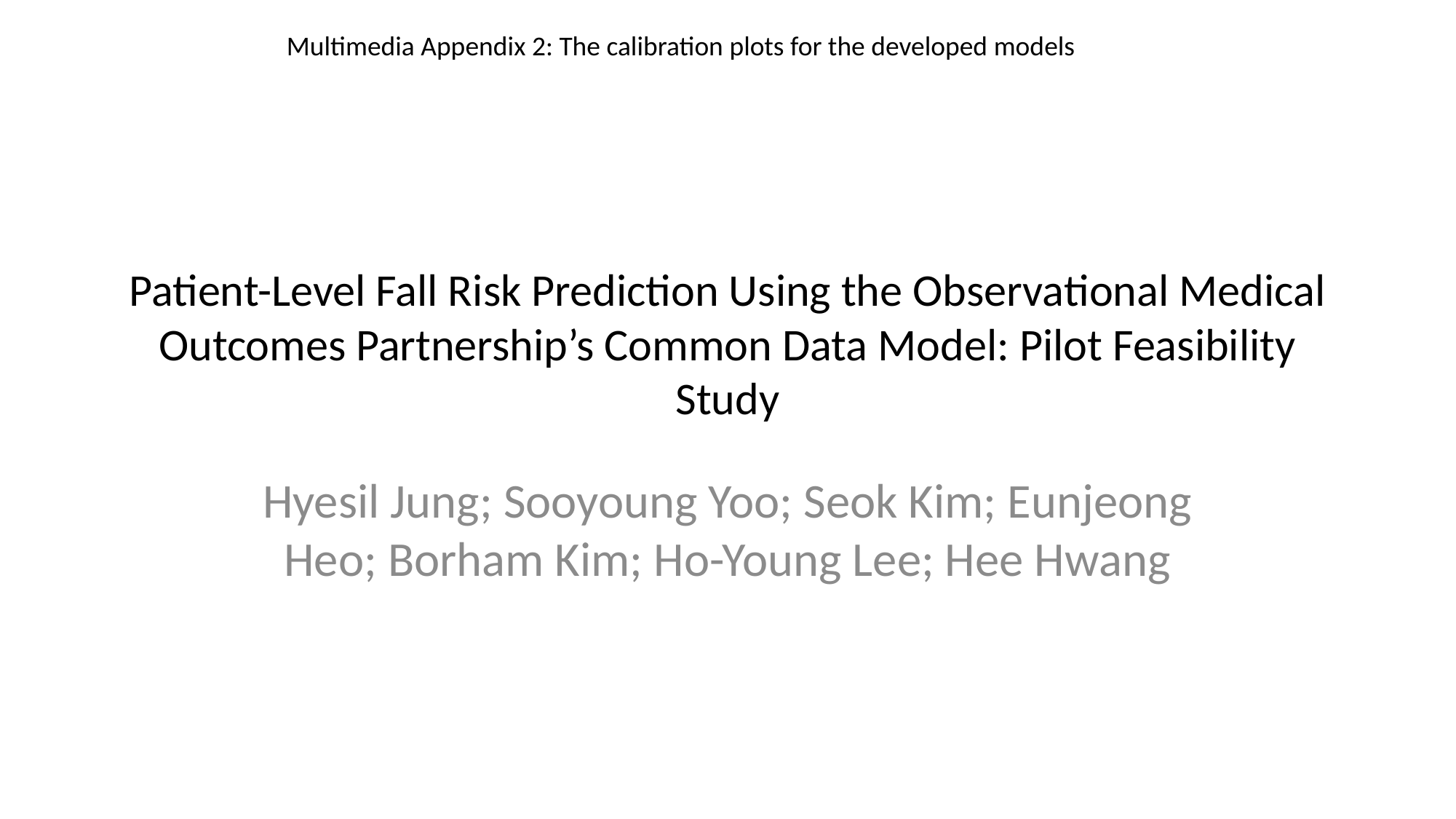

Multimedia Appendix 2: The calibration plots for the developed models
# Patient-Level Fall Risk Prediction Using the Observational Medical Outcomes Partnership’s Common Data Model: Pilot Feasibility Study
Hyesil Jung; Sooyoung Yoo; Seok Kim; Eunjeong Heo; Borham Kim; Ho-Young Lee; Hee Hwang

## Slide 2
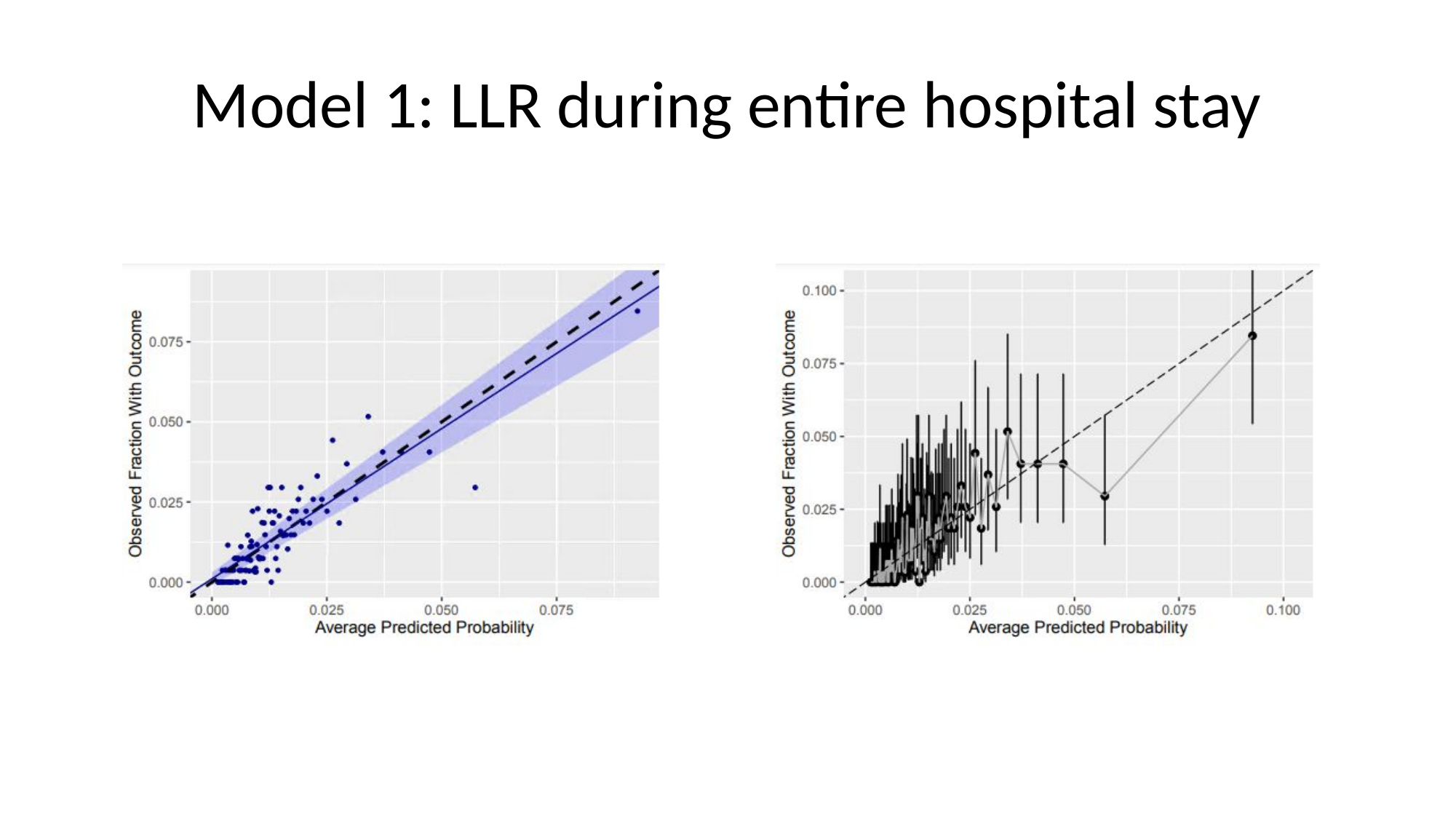

# Model 1: LLR during entire hospital stay

## Slide 3
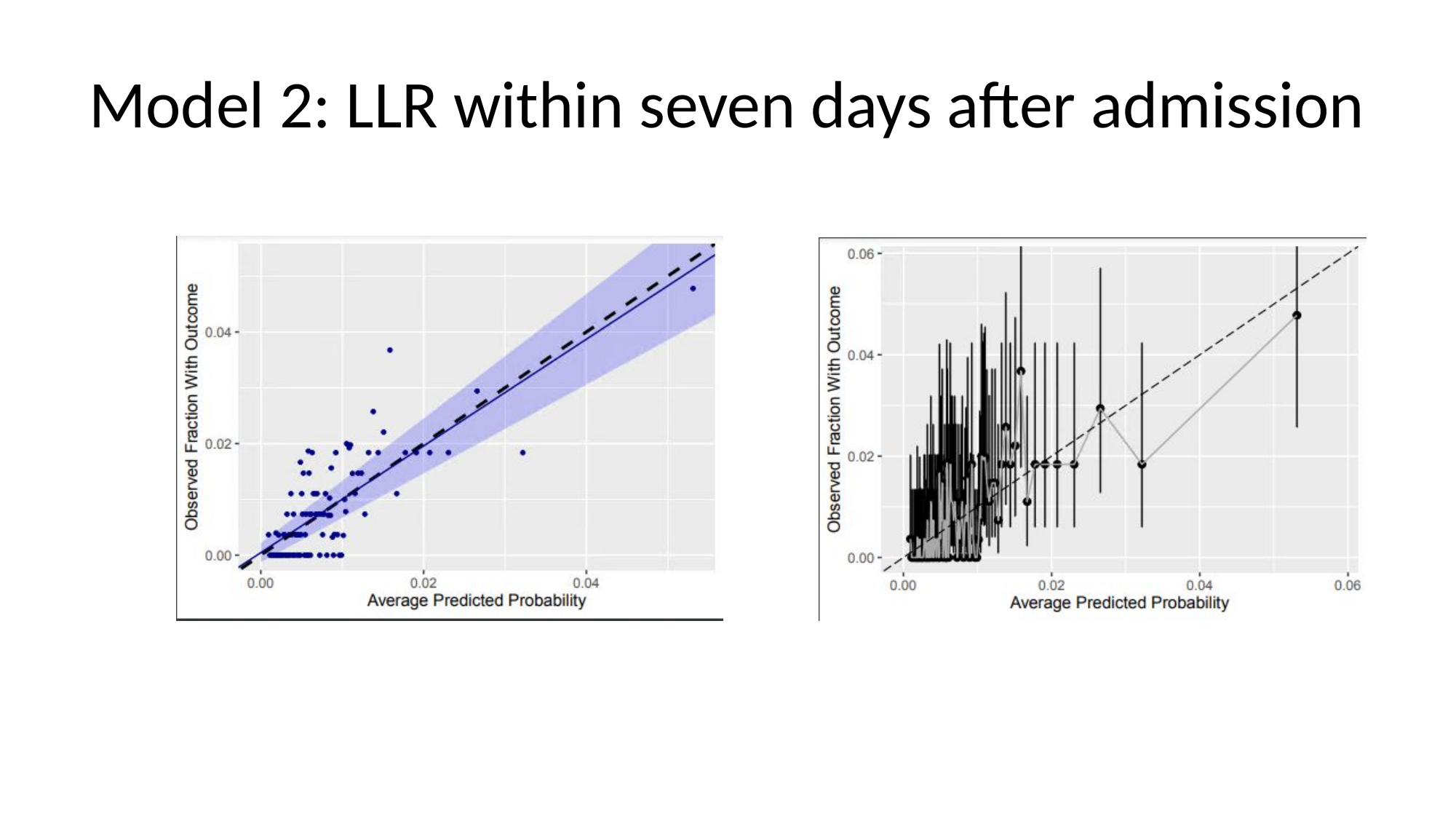

# Model 2: LLR within seven days after admission

## Slide 4
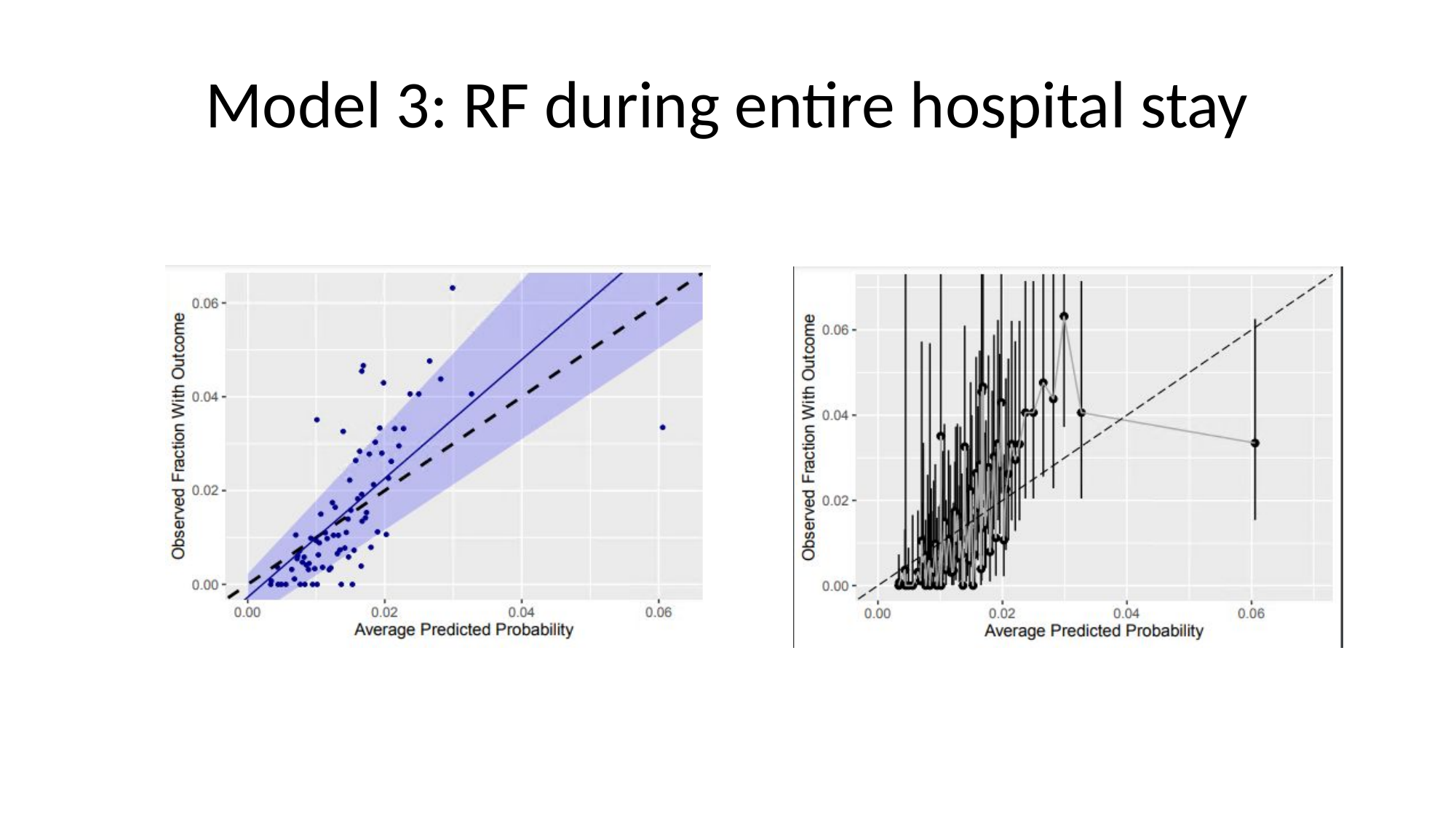

# Model 3: RF during entire hospital stay

## Slide 5
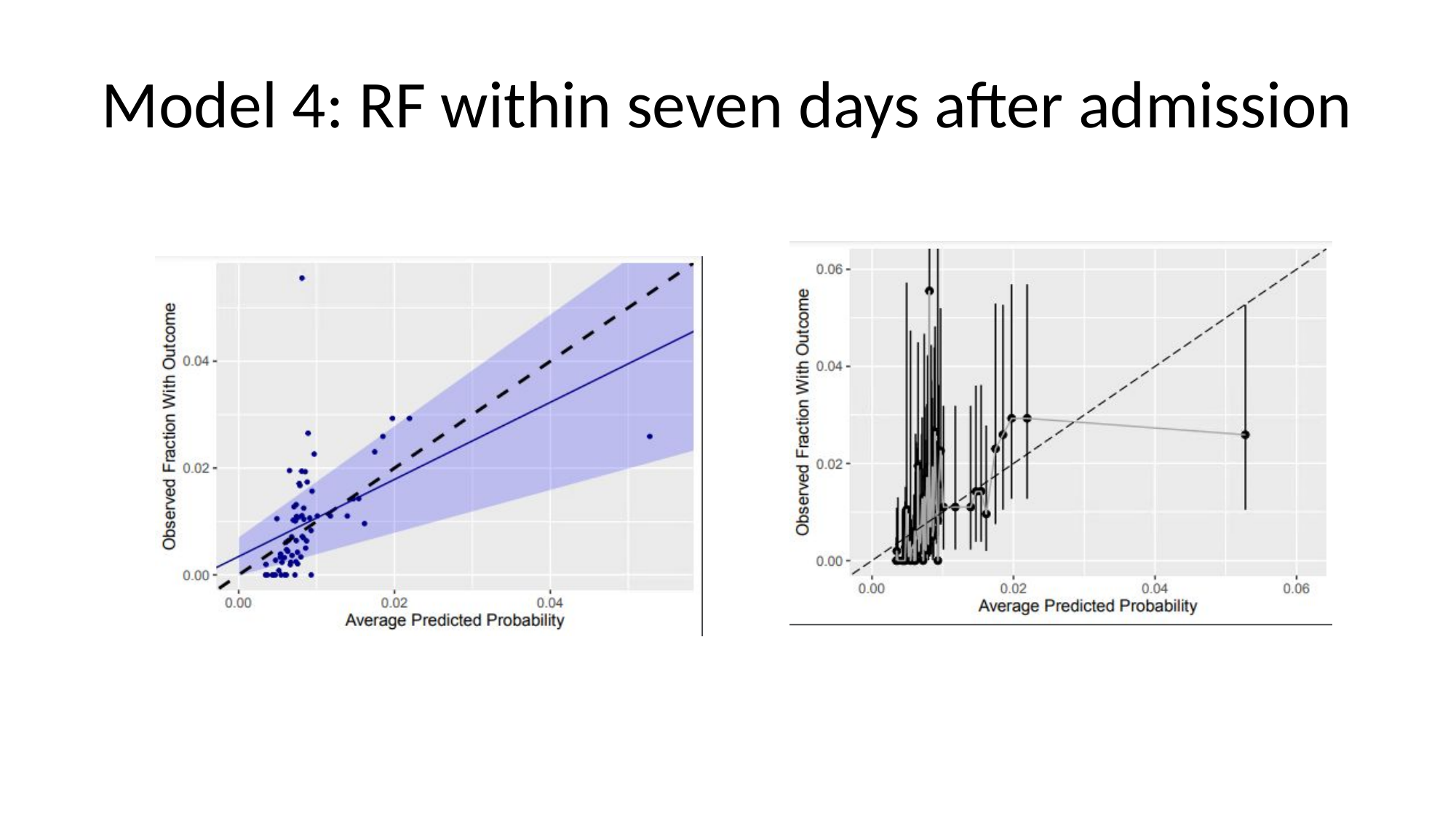

# Model 4: RF within seven days after admission
